# Beyond the lab coat: methodological challenges in space life sciences

**DOI:** 10.3389/fphys.2025.1663701

**Published:** 2025-10-03

**Authors:** Martine Van Puyvelde, Nicholas H. van den Berg, Lara Stas, Perseverence Savieri, Hortense Corlùy, Jeroen Van Cutsem, Xavier Neyt, Guido Simonelli, Nathalie Pattyn

**Affiliations:** ^1^ VIPER Research Unit, LIFE department, Royal Military Academy, Brussels, Belgium; ^2^ Brain, Body and Cognition, Faculty of Psychology and Educational Sciences, Vrije Universiteit Brussel, Brussels, Belgium; ^3^ School of Natural Sciences and Psychology, Faculty of Science, Liverpool John Moores University, Liverpool, United Kingdom; ^4^ Center for Advanced Research in Sleep Medicine, Hôpital du Sacré-Coeur de Montréal, CIUSSS du Nord de l’Île-de-Montréal, Montreal, QC, Canada; ^5^ Core Facility-Support for Quantitative and Qualitative Research, Vrije Universiteit, Brussels, Belgium; ^6^ Faculty of Medicine, Research Center for Digital Medicine Research Group, Vrije Universiteit, Brussels, Belgium; ^7^ Human Physiology and Sports Physiotherapy Research Group, Vrije Universiteit Brussel, Brussels, Belgium; ^8^ Department of Medicine, Faculty of Medicine, Université de Montréal, Montreal, QC, Canada; ^9^ Department of Neuroscience, Faculty of Medicine, Université de Montréal, Montreal, QC, Canada

**Keywords:** space life sciences, space analogs, field research, research methodologies, space, space methodology

## Abstract

As plans for deep space and long-duration missions advance, research in space and space-analog environments is becoming an urgent scientific priority. However, this type of fieldwork poses a unique set of challenges. The development of research methodologies and designs cannot rely on broad evidence base and thus requires scientific judgment and multidisciplinary psychophysiological expertise. Most studies comprise small samples, often lack control groups, sex differences have seldom been directly tested in this area and inter-individual variability is prevalent in this population. Moreover, this research domain is characterized by several exceptional factors that must be addressed. The target population is highly trained and not representative of the general population, demanding adapted study designs and highly sensitive and operationally relevant research tools. To avoid overburdening the already heavy operational schedules of this population, a careful and feasible balance must be established between scientific data quality and acceptable monitoring load. Furthermore, several issues of location, timing, and type of baseline measures must be explicitly considered, while long-term follow-up designs are necessary to assess both recovery and persistent post-mission effects. Major space agencies have indeed identified methodological issues as a knowledge gap in this area. In this review, we provide an overview of these methodological challenges unique to space life sciences and offer solutions where possible. We argue that space research remains feasible despite these constraints, but only when it is approached with the understanding that such fieldwork often requires fundamentally different methods than traditional laboratory science.

## Introduction

Humanity is increasingly aiming for a long-term presence in space. To support this goal, space life sciences encompass multiple research domains, each contributing to our understanding of human adaptation. As recently summarized (Berliner et al., 2024), three major areas intersect here. First, technological innovations to support space travel; second, environmental research on potentially sustaining future life; and third, studies on the physiological and psychological effects of spaceflight—covering the so-called “human factor”. In the context of the current article, the human factor refers to the adaptability of the atypical, highly trained operator who typically performs above average across all performance domains (Strangman et al., 2014).

The major space agencies have each defined research roadmaps providing a comprehensive overview of knowledge gaps (e.g., European Space Agency, 2016). The current review aims at identifying the unique methodological challenges posed by the space environment which need to be addressed to close those knowledge gaps. These challenges may affect every phase of research—from design over field data collection to final statistical analysis and interpretation. This methodological perspective thus identifies recurring pitfalls and highlights proposed solutions. Hence, this is not an exhaustive review, but a summary of key methodological patterns and constraints observed in space (-analog) studies.

### Sampling challenges in space research: small samples, sex differences, inter-individual variability and the absence of control groups

#### Small sample sizes

The NASA Apollo Biomedical Results Report (1974) stated: ‘… because of the small number of individuals who flew in space and because of the variability of their responses, it was impossible to distinguish between space-related physiological changes and individual physiological variations (Johnston and Dietlein, 1974, p.43)’.

To date, little has changed. The literature on space-related research remains saturated with concerns regarding small sample sizes that are statistically underpowered, inter-individual variability that hinders distinction between environmental- and individual-related factors and the absence of control groups (e.g., Clément, 2025; Desai et al., 2022; Mairesse et al., 2019; Pattyn et al., 2009; Stavnichuk et al., 2020; Strangman et al., 2014). This raises the question as to how to address the problem.

A possible solution is to promote international cooperation by standardizing protocols across space agencies, facilitating data sharing, and enabling joint analyses across missions (Desai et al., 2022; Roberts et al., 2020; Stavnichuk et al., 2020; Van Ombergen et al., 2022). Similarly, some authors pooled blood sample data (e.g., Bisserier et al., 2021; Brojakowska et al., 2022) or densitometry results (Sibonga et al., 2015) across ISS and MIR missions.

Molecular biology seemed like a way around the small number of individuals. This includes cross-species cell analyses, development of predictive models, real-time monitoring, and standardization of crew dose and risk metrics related to cosmic radiation (Slaba et al., 2025; Willis et al., 2024). For instance, Galčenko et al. (2025) used transcriptomic data from human cell lines exposed to microgravity and Michaletti et al. (2017) examined osteoblasts from three healthy hip-replacement donors. However, even here, Stavnichuk et al. (2020) reported high variability in bone formation markers and emphasized that more data are needed to determine to what extent individual covariates (e.g., age, physical activity, nutrition) may influence outcomes.

#### Sex differences

Regarding sex-differences, space research data are even scarcer. Earth-based evidence shows differences in sleep, activity, and cognition—domains critical for mission success. Women generally sleep longer but experience more wake after sleep onset (WASO) and insomnia (Jonasdottir et al., 2021). In a hypoxic bedrest space analog, the return of WASO-deviations to baseline was absent in females (Van Cutsem et al., 2022). During Antarctic overwintering, only men showed activity decline, but women reported more sleep and psychosocial disturbances (Steinach et al., 2016). Cognitively, women prioritize accuracy over speed and men vice versa—indicating complementary strengths (Hughes-Fulford et al., 2024; Mark et al., 2014). Though more prone to motion sickness, women may outperform men in vestibular tasks, albeit with higher variability (Zhang et al., 2024).

In space, female astronauts showed higher rates of immediate post-flight orthostatic intolerance—the inability to remain upright without fainting—and greater plasma volume loss (Mark et al., 2014). Some authors suggest that radiation exposure limits are lower for women (e.g., Mark et al., 2014; Parihar et al., 2020). Likewise, rodent studies indicated that radiation may threaten cognition through neuroinflammation and hippocampal damage (Krukowski et al., 2018). On the other hand, Hughes-Fulford, 2023a emphasized that there is no clear evidence that women are at higher risk of radiation-related effects during the mission, only that postflight cancer risk may reduce their lifespan by 3%. Nevertheless, one fundamental difference between men and women is that women “carry” all their gametes at all times, potentially increasing the risk of radiation-related effects on future offspring.

To address these problems, researchers propose to increase female participation in spaceflights, in order to improve our understanding and move beyond the default male model (D’souza et al., 2022; Mark et al., 2014). For instance, when it concerns countermeasures, an increased sex-disaggregated approach could lead to more adjusted female space health and security measures for women in space.

#### Statistical and methodological considerations

When studying small populations, researchers face profound statistical challenges that demand tailored approaches different from conventional inferential methods. In such contexts, classical hypothesis testing becomes underpowered, *p-*values unstable, and models may fail to converge, while the power and generalizability of findings remain limited (Pattyn et al., 2009). Moreover, traditional techniques such as repeated-measures ANOVA can exacerbate the problem by applying listwise deletion, thereby further reducing statistical power. Corrections for multiple comparisons, such as Bonferroni adjustments, are also overly conservative in this context, increasing the risk of Type II errors (i.e., not finding a true effect).

Therefore, tailored analytical approaches are needed. A key distinction must be made between studies that describe the entire astronaut population and those that collect a sample from a wider reference population. In the first case, when all individuals in the target group are observed (e.g., all active astronauts on a certain mission), statistical inference is unnecessary—here, descriptive statistics, individual-level analyses, and visualizations suffice. However, if the goal is to generalize to a broader population (e.g., astronauts across agencies or future crews) statistical inference becomes necessary and must be adapted to small-sample limitations.

In this context, researchers are encouraged to focus on effect sizes, and the uncertainty of parameter estimates via confidence intervals. Effect sizes quantify the magnitude of observed effects. For example, effect sizes can reflect the strength of physiological changes in response to spaceflight. Confidence intervals indicate estimate precision with wider intervals signalizing greater uncertainty.

A helpful analytical approach in small sample studies might be using Bayesian methods. They allow the incorporation of prior knowledge (e.g., from analog populations or historical missions) to stabilize estimates and improve inference (Gelman and Shalizi, 2013; McElreath, 2020). Also, hierarchical (multilevel) models can be considered useful, as they can borrow strength across repeated measures or related individuals (e.g., astronauts in the same space shuttle) to enhance power and account for nested data structures. These models naturally accommodate missing data under more realistic assumptions (e.g., missing at random), making them more appropriate than techniques like repeated-measures ANOVA. They also allow researchers to model individual differences explicitly. This is particularly useful because intra-individual variability often exceeds between-group differences (e.g., sex-differences) in astronaut research (Robin et al., 2023).

Lastly, to eliminate the effect of confounding variables in the context of limited power, Pattyn et al. (2009) suggested comparing data from individual astronauts to carefully matched control groups (matching based on gender, background variables and other relevant features).

#### Absence of control groups

Control groups have only become common in recent studies. Yet, without control groups, distinguishing environmental from individual factors remains difficult. Matching for the multiple stressors faced in space is not evident (Desai et al., 2022), but some innovative approaches have been suggested. Pattyn et al. (2009) matched astronauts with similar control groups to apply neuropsychological analyses of cognition in flight. To study radiation effects, Boice (2019) proposed examining cognitive performance in nuclear workers with high radionuclide exposure with astronaut-standardized tasks. The NASA Twins Study (Garrett-Bakelman et al., 2019) compared an astronaut in space with his identical twin on Earth and Moore et al. (2019) used a matched ground-control group and a sleep-restricted cohort. Bosch-Bruguera et al. (2021) also used a matched control-group in a longitudinal design to account for maturation effects of their investigation of skill decay over time during an Antarctic overwintering. Moreover, as will be discussed below, the use of control groups has already yielded new insights into cognitive impairment in space that were previously not available (e.g., Kuldavletova et al., 2023; Moore et al., 2019; Pattyn et al., 2009; Stahn et al., 2019). Therefore, authors are encouraged to invest time and resources in research designs implementing control groups.

### Measurement validity under operational constraints: from test batteries to wearables under controlled conditions

#### Sensitivity and accuracy of a standardized cognitive test battery adapted to space operationality: how to measure a rigorously trained population

Given the exceptional cognitive profile of astronauts, current test batteries may lack the sensitivity to detect subtle impairments (Fowler and Manzey, 2000; Pattyn et al., 2009; Strangman et al., 2014; Van Puyvelde et al., 2022a) as well as tasks capturing the operational relevance critical for mission success (Moore et al., 2019; Petit et al., 2019; Wenzel, 2021). Although many studies reported no cognitive decline or even improvement during missions (e.g., Dev et al., 2024; Garrett-Bakelman et al., 2019; Slack et al., 2016; Paul et al., 2010), other findings—including both anecdotal descriptions such as self-reports and interviews (e.g., Bluth, 1984; Johnston and Dietlein, 1974; Burgess, 2000; Manzey et al., 1995; Van Puyvelde et al., 2022b) and studies using operational tasks, control groups and brain references—found impairments that persisted, even after return to Earth (e.g., Clement, 2025; Kuldavletova et al., 2023; Moore et al., 2019; Pattyn et al., 2009; Jones et al., 2022; Petit et al., 2019; Stahn et al., 2019). Therefore, the observed improvements in certain studies might reflect learning or observer effects (e.g., Clément, 2025; Desai et al., 2022; Strangman et al., 2014) rather than true enhancement. Indeed, Benke et al. (1993) used 30 habituation sessions over 8 months pre-flight to avoid learning effects in-flight. However, aiming for maximal stabilization of performance also means losing sensitivity to environmental or situation influences, trade-off performance scientists are well aware of (Pattyn et al., 2024). Accordingly, Wenzel (2021) emphasized the need to define clear thresholds for acceptable versus unacceptable performance. This implies that both baselines and normative criteria must be tailored to specific tasks to determine when performance has significantly declined.

#### Sensitivity and accuracy of wearables: how to balance monitoring load and scientific data quality

Survey fatigue and monitoring burden remain key challenges in every type of fieldwork including space (-analog) research (Ghafourifard, 2024; Andreassi, 2007; Kelly et al., 2005), especially when workload, sleep loss, and frustration start to accumulate (Pattyn et al., 2018; Van Puyvelde et al., 2022a). Therefore, finding a balance between data-collection quality and practical feasibility in terms of crew’s preference and environmental constraints is critical. Wearables are gaining popularity as practical, user-friendly alternatives (Fonseca et al., 2017) despite the lack of a robust multistage validation (Doherty et al., 2024; Giurgiu et al., 2023); hence, blurring lines between commercial and scientific use (Baron et al., 2018; Lee and Finkelstein, 2015).

This search for balance between the scientific ideal and environmental realities may explain why Jones et al. (2024) used the Apple Watch Series 6 to assess heart rate variability—despite previous studies showing its limited accuracy even in resting position (e.g., Bonneval et al., 2025; O’Grady et al., 2024), let alone in dynamic operational settings. Similarly, the choice to use 1-h HRV windows rather than the 3–5 min guidelines (non-stationarity, Berntson et al., 1997) and omitting raw signal quality checks draws the attention to the importance of ensuring that practicality does not come at the expense of scientific and physiological expertise.

### Baseline? the importance of longitudinal investigations

Multiple stressors may elicit distinct physiological or cognitive responses (Wenzel, 2021). The timing, location, and type of baseline measures must therefore be chosen carefully. Longitudinal time-points can robustly build a reference framework for each participant. For instance, physiological stress research distinguishes between phasic (acute) and tonic (anticipatory) stress responses—such as pre-mission logistical or personal concerns—which can confound both baseline and subsequent in-flight measures (Pattyn et al., 2009; Van Puyvelde et al., 2020). Such baseline distortions may help explain reported in-flight cognitive improvements. Indeed, a closer look at the TWIN Study (Garrett-Bakelman et al., 2019) results reveals conspicuously low baseline levels compared to the early in-flight performance. Besides timing, baseline locations must also be consistent or contextually meaningful (Bialeschki et al., 2012). Overall, based on psychophysiological research, three types of baselines are recommended: (1) a resting baseline to compare with resting in-flight measures, (2) a “vanilla” baseline (i.e., a neutral task matched in sensory/motor load to the experimental task), to isolate metabolic effects inherent to the imposed task (Tininenko et al., 2012), and (3) a task-specific physiological baseline to compare with in-flight measures across conditions.

Careful timing is also crucial for in-flight measures, as most adaptation timelines for basic physiological processes still need to be defined. Entering space was described as a “traumatic experience of habituative adaptation,” sometimes reducing workload capacity (Johnston and Dietlein, 1974, p. 850). Despite decades of technological evolution, this description from the heroic age still stands. Similar habituation periods have since been observed in space (-analog) research (Pattyn et al., 2018; Clément et al., 2020). For instance, polar studies suggest a three-week adaptation period—challenging Jones et al. (2024) assumption that cognitive space-induced effects are fully observable on day 1 rather than day 4.

Finally, post-flight measures are important for assessing recovery and long-term effects. Several studies have documented persistent post-flight impairments (e.g., Garrett-Bakelman et al., 2019; Manzey et al., 1995; Moore et al., 2019), including altered gene expression, DNA damage, telomere shortening, and cognitive deficits. Autonomic regulation changes are even shown to last longer in the post-flight recording than the actual in-flight exposure (Migeotte et al., 2003). At the skeletal level, bone density recovery often remained incomplete, even 2 years postflight (Sibonga et al., 2020; Vico et al., 2017). These findings—along with interviews from analog environments (Van Puyvelde et al., 2022b)—highlight the need for protective strategies; not only during but also after missions. One proposed solution is to continuously measure until recovery has occurred (Roberts et al., 2020; Wenzel, 2021).

### Time-in-space or time-on-station

Long-duration space research remains limited, despite its growing relevance. The scarcity of such missions blurs the definition of what is “long-duration”, which may lead to the classification of relatively short flights as “long-duration. To illustrate, in a space context, Strangman et al. (2014) considered space missions over 21 days as “long-duration,” reflecting this lack of longer flights. In contrast, in a space-analog context, Van Puyvelde et al. (2022b) defined missions over 12 months as “long-duration”. Today, aside from the Apollo notebooks, only two major long-duration studies have been published: the 340-day ISS Twin Study (Garrett-Bakelman et al., 2019) and the 438-day MIR mission (Manzey et al., 1998).

Anyhow, prolonged missions expose astronauts to sustained cumulative stressors—gravity shifts, radiation, sleep loss, fatigue and workload variations—all of which may, at some point, override psychophysiological compensation mechanisms. Several effects are time-dependent. For instance, bone resorption markers (bone loss) peaked within 11 days, while formation markers responded too slowly and weakly to reverse damage. After longer stays, breakdown decelerated faster, but the damage was greater and recovery remained incomplete after 3–5 months (Stavnichuk et al., 2020). Sibonga et al. (2024) similarly reported greater bone loss after longer missions, with models estimating that 62% of astronauts would return from a Mars mission with osteoporosis-level T-scores (Axpe et al., 2020).

Long-duration space (-analog) missions also affect hippocampal regions involved in memory, emotion, and spatial cognition (Stahn et al., 2019; Stahn and Kühn, 2021). Brain imaging shows structural changes, including brain shift, cerebrospinal fluid redistribution, ventricular expansion, and gray matter loss (e.g., McGregor et al., 2023; Van Ombergen et al., 2018; 2019). Ventricle expansion also increases with mission length, with the greatest changes in the first 6 months and up to 3 years before full recovery (McGregor et al., 2023). Petit et al. (2019) observed attention lapses, reflected in theta oscillations during electroencephalographic recordings, along with impaired visuomotor performance during docking tasks in an ISS crew after 2 months in space.

According to Clement (2025), the accumulation of stressors over time may underlie the often reported “space fog”. Jones et al. (2022) supported this view, noting, however, that sleep quantity was a defining factor in the multi-stressor dynamics of neurobehavioral responses and perceived workload over time. Moreover, radiation-induced cognitive deficits are shown to worsen under high workload—even at low exposure levels (Hanbury et al., 2016). Hence, the combined burden of time-in-space and workload variations may deplete cognitive reserves and/or increase the risk of relying on pharmacological support (Johnston and Dietlein, 1974; Strangman et al., 2014; Van Puyvelde et al., 2022a; Van Puyvelde et al., 2022b).

Overall, time-in-space must be systematically included in multi-stressor research, as its cumulative burden on cognitive, skeletal, and neural health is critical for future long-duration missions. This means that gathering meta-data about the multiple dynamics of the context and their stressors (unexpected events and logbooks included) is essential in order to better understand and interpret study results.

### Anonymization

In qualitative research, the trade-off between providing sufficient detail to address research questions and protecting participant anonymity has long been acknowledged (e.g., Kaiser, 2009). Similar ethical and methodological challenges arise in space (-analog) research. Due to the small number of crew members and the public and media attention generated by such missions, full anonymization is often unfeasible. As a result, logbook reports describing impactful events that are—as described above—potentially critical for interpreting unexpected outcomes, may be excluded from analysis. This limitation has already been cited as a reason why certain data remained inaccessible or unavailable for desired long-term follow-up analyses (e.g., Bisserier et al., 2021; Jones et al., 2024).

### Low earth orbit (LEO) and beyond LEO missions: location-specific variation in the impact of gravity and ionizing radiation (IR)

Except for the Apollo notebooks, most of the studies are limited to low Earth orbit (LEO) missions. Yet, both gravitational and radiation exposure effects vary with the trajectory and destination. For instance, a Mars mission involves a transition from Earth’s gravity (9.807 m/s^2^), through microgravity in transit, to Mars’ reduced gravity (3.711 m/s^2^), illustrating location-specific gravitational shifts (Bettiol et al., 2018).

Similarly, Moon IR-levels can double those on the ISS and reach 200–1000 times Earth-levels (Asrar, 2025; Zhang et al., 2020). ESA estimated that a Mars mission could expose astronauts in 1 day to the equivalent of a full year’s radiation on Earth—and this repeatedly for months (Asrar, 2025). Astronauts face both acute bursts (e.g., EVAs or solar storms) and prolonged exposure (Tavakol et al., 2024). IR thus remains a key risk, and both journey and destination must be included in estimation models (Willis et al., 2024).

## Discussion

Space (-analog) research faces several unique methodological challenges including small and heterogeneous samples, inconsistent baselines and lack of tools tailored to highly trained astronauts (Desai et al., 2022; Strangman et al., 2014). These limitations worsen when sex differences are ignored (Hughes-Fulford, 2023a). Although advances like omics modeling, biosample analyses, and cross-agency data harmonization (Abdelfattah et al., 2024; Galčenko et al., 2025; Roberts et al., 2020) are promising, their ecological validity remains limited. Nonetheless, limited data is already guiding policy. For instance, a recent model indicated an 85.2% chance for female vs 22.8% for male astronauts to meet anxiety criteria during Mars missions (Desai et al., 2022)—an aspect that the authors indicated as a comorbidity factor in sleep problems. Such interpretations, while well-intended, risk overgeneralization.

Earth-based matched control groups remain underused, likely due to logistical and financial constraints (e.g., Boice, 2019; Kuldavletova et al., 2023). Yet funding should account for these essential but costly designs. Data repositories of major space agencies have been in the making for decades but are still not enforced. Moreover, inconsistent baselines, follow-up, and recovery measurements risk distorting data (Migeotte et al., 2003; Sibonga et al., 2020; Vico et al., 2017). Long-duration missions are particularly hazardous due to cumulative stressors like microgravity, radiation, sleep loss and workload variations (Hanbury et al., 2016; Moore et al., 2019; Van den Berg et al., 2023). Therefore, extended follow-up studies to monitor post-mission recovery and long-term health outcomes are warranted (Roberts et al., 2020; Sibonga et al., 2020; Vico et al., 2017).

Fieldwork is demanding, time-consuming, and often requires methodological deviations from traditional lab-based research. To gather statistically powered field samples remains difficult—especially for sex-specific comparisons. For instance, our team needed 14 years and seven Antarctic overwintering data collection campaigns to collect an acceptable database of 30 female winter-over sojourners in sleep studies using polysomnography. Such timelines are unsustainable for most research units considering the pace of research funding and required publishing.

Hence, more resources and innovative statistical approaches are needed. Considering the methodological realities discussed, relying solely on traditional statistical metrics such as *p*-values remains insufficient, especially given the small sample sizes and pronounced individual variability characteristic of space (-analog) research. Enhanced reporting practices, including effect sizes, comprehensive visualizations, and precise parameter estimations via confidence intervals, should thus be prioritized. Advanced statistical methods, notably multilevel and mixed-effects models, further align methodological rigor with the inherent complexity of space-based research.

We therefore strongly encourage authors to explicitly state in their publications that fieldwork—especially in extreme environments—demands a fundamentally different methodological approach than traditional laboratory studies. Only by acknowledging these realities, can we ensure that field studies are evaluated fairly, appreciating their unique contextual, logistical, and scientific contributions rather than penalizing inherent constraints. This would avoid the “dormant data” that currently plagues the field of space life sciences, where relevant measurements are sometimes never published due to their anecdotal nature, which is not familiar to reviewers not specialized in this area of expertise.
